# Annexin A1 peptide Ac2-26 mitigates ventilator-induced lung injury in acute respiratory distress syndrome rats and partly depended on the endothelial nitric oxide synthase pathway

**DOI:** 10.1590/acb371203

**Published:** 2023-01-13

**Authors:** Yingnan Ju, Xikun Sun, Guangxiao Xu, Qihang Tai, Wei Gao

**Affiliations:** 1 MD. Harbin Medical University – Department of Intensive Care Unit – Third Clinical College – Harbin, China.; 2 MS. Harbin Medical University – Department of Anesthesiology – The Second Affiliated Hospital – Harbin, China.; 3 MS. Harbin Medical University – Department of Anesthesiology – The Second Affiliated Hospital – Harbin, China.

**Keywords:** Annexin A1, Ventilator-Induced Lung Injury, Respiratory Distress Syndrome

## Abstract

**Purpose:**

Although mechanical ventilation is an essential support for acute respiratory distress syndrome (ARDS), ventilation also leads to ventilator-induced lung injury (VILI). This study aimed to estimate the effect and mechanism of Annexin A1 peptide (Ac2-26) on VILI in ARDS rats.

**Methods:**

Thirty-two rats were randomized into the sham (S), mechanical ventilation (V), mechanical ventilation/Ac2-26 (VA), and mechanical ventilation/Ac2-26/L-NIO (VAL) groups. The S group only received anesthesia, and the other three groups received endotoxin and then ventilation for 4 h. Rats in the V, VA and VAL groups received saline, Ac2-26, and A c2-26/N5-(1-iminoethyl)-l-ornithine (L-NIO), respectively.

**Results:**

All indexes deteriorated in the V, VA and VAL groups compared with the S group. Compared with V group, the PaO_2_/FiO_2_ ratio was increased, but the wet-to-dry weight ratio and protein levels in bronchoalveolar lavage fluid were decreased in the VA group. The inflammatory cells and proinflammatory factors were reduced by Ac2-26. The oxidative stress response, lung injury and apoptosis were also decreased by Ac2-26 compared to V group. All improvements of Ac2-26 were partly reversed by L-NIO.

**Conclusions:**

Ac2-26 mitigates VILI in ARDS rats and partly depended on the endothelial nitric oxide synthase pathway.

## Introduction

Acute respiratory distress syndrome (ARDS) is a sudden and serious lung injury in the intensive care unit (ICU), which is characterized by deterioration of alveolar-vascular permeability and pulmonary interstitial edema, pulmonary focal lesions or systemic inflammation and severe hypoxemia^1^. The prevalence of mild, moderate and severe ARDS was 30, 46.6, and 23.4%, respectively, reported in a recent epidemiological analysis^2^. Moreover, 39% of patients in the ICU received mechanical ventilation (MV) support to maintain oxygenation^3^. However, MV not only provides respiratory support, but also results in lung injury, which was named VILI^4^. Evidence suggests that imbalanced inflammation plays a key role in ARDS and VILI^1^^,^ 5. The ventilation for pre-inflammatory lung tissue of ARDS could result in overstretching of the alveoli, activating inflammatory cells and releasing proinflammatory factors and further damaging the pre-injured lung^6^ and resulted to lung edema and severe hypoxemia^7^^,^ 8. Therefore, effective control of inflammation is a potential strategy to prevent VILI in ARDS.

Annexin A1 (AnexA1) is an endogenous glucocorticoid-regulated anti-inflammatory protein and was shown to suppress inflammation. Its N-terminal-derived peptide Ac-ANX-A1 (Ac2-26) had been indicated to reduce lung injury via activation of formyl peptide receptor and endothelial nitric oxide synthase (eNOS) pathway^9^^,^ 10. Considering the role of eNOS in acute lung injury and VILI^11^^,^ 12, and the regulation of Ac2-26 and eNOS^13^^,^ 14, we speculated that the Ac2-26 reduces VILI in ARDS rats partly via the eNOS pathway.

## Methods

The approval protocol number of the Animal Research and Care Committee of Harbin Medical University is SYDW2022-095.

### Study design

All the procedure of this study was approved by the Animal Research and Care Committee of Harbin Medical University. Thirty-two Sprague Dawley rats which were 13 weeks old and had body weights of 300 ± 20 g were randomized into four groups: the sham group (S), mechanical ventilation group (V), MV/Ac2-26 group (VA) and MV/Ac2-26/L-NIO group (VAL) (n = 8). All rats received anesthesia with 3% pentobarbital sodium (30 mg/kg intraperitoneal injection). After anesthesia and local infiltration of lidocaine, the caudal artery and vein were cannulated to analyze the arterial blood gas analysis, blood samples were collected, and saline was injected.

The rats in the S group only received anesthesia and intubation. The rats in the V, VA and VAL groups first received an intravenous injection of endotoxin (Sigma-Aldrich, St, Louis, MO, United States of America) to simulate ARDS^15^. After 30 min of injection with endotoxin, the rats were analyzed for arterial blood gas to detect the PaO_2_/FiO_2_ ratio. ARDS establishment was judged to be successful when the PaO_2_/FiO_2_ ratio was less than 300^1^. Then, these rats received a large tidal volume of MV for 4 h to induce VILI. During MV, the tidal volume was set to 30 mL/kg according to previous study^16^, the respiratory rate was set to 50 min, and the inspiratory/expiratory ratio was set to 1:1 without positive end-expiratory pressure (PEEP).

Based on results of previous studies, the dosage of Ac2-26^10^ and inhibitor of eNOS^17^^,^ 18 were confirmed at 1 and 10 mg/kg. At initiation of MV, the saline, Ac2-26 (1 mg/kg) (Sigma, United States of America) and Ac2-26 (1 mg/kg) combined with N5-(1-iminoethyl)-l-ornithine (L-NIO) (Santa Cruz) (10 mg/kg)^17^^,^ 18 were injected into the rats in the V, VA and VAL groups immediately. During the MV procedure, the anesthesia was maintained with 3% pentobarbital sodium (10 mg/kg) and 0.6 mg/kg rocuronium per hour.

### Randomization

Thirty-two number from 1 to 32 were labeled in 32 cards, which were stored in an envelope. The numbers 1 to 8, 9 to 16, 17 to 24, and 25 to 32 were allocated into S, V, VA and VA/L group, respectively. The rat was randomly selected by a card after anesthetized and allocated into S, V, VA or VA/L group according to the number on the card.

### Sample collection

The peripheral blood and arterial blood analyses were conducted at baseline, 30 min after injection of endotoxin, and 4 h after ventilation. All rats were sacrificed with an overdose of anesthetics after 4 h of ventilation, and the lung tissues and blood were collected. The right lung tissue was collected to analyze lung injury, apoptosis, and protein analysis. The left lung was injected with saline to collect the bronchoalveolar lavage fluid (BALF). The serum and BALF were centrifuged at 1,000 g at 4 °C for 15 min, and the supernatants were stored at -80 °C for further analysis. After the procedure, rats were injected with large doses of anesthetic drugs.

### The alveolar-capillary permeability

We performed the arterial blood gas analysis with a Rapidlab 348 system (Bayer Diagnostics, Germany) to calculate the PaO_2_/FiO_2_ ratio. Moreover, we also detected the protein concentration and lung tissue wet/dry weight ratio. Part of the right upper lung tissue of all rats was collected to weigh, and then this lung tissue was dried at 60 °C for 48 h. The weight ratio of wet lung tissue to dry lung tissue was calculated. We also tested the protein levels of BALF using the BCA method. These data were calculated to evaluate the effect of AnexA1 on pulmonary alveolar-capillary permeability.

### Local and systemic inflammation analysis

To evaluate the effect of AnexA1 on systemic and local inflammation in VILI, we collected peripheral blood samples and BALF. The inflammatory factors, including tumor necrosis factor-α (TNF-α), interleukin-1β (IL-1β), interleukin-6 (IL-6) and interleukin-10 (IL-10), in peripheral blood and BALF were detected with enzyme-linked immunosorbent assay (ELISA) kits (Wuhan Boster Bio-Engineering Limited Company, Wuhan, Hubei, China). The intercellular adhesion molecule 1 (ICAM-1) and interleukin-8 (IL-8) levels in serum were also estimated. Furthermore, after centrifugation, BALF deposits were stained with Giemsa by an independent pathologist to count the number of macrophages and neutrophils.

### Oxidative stress response

We collected part of the right lung tissues and homogenized them with saline. The homogenate was centrifuged, and the homogenized supernatants were collected to analyze the malondialdehyde (MDA) concentration and the myeloperoxidase (MPO) and NADPH activities by using specific kits (Nanjing Jiancheng).

### Histopathologic lung injury evaluation

We analyzed lung tissue histological injury with hematoxylin and eosin (HE) staining. First, part of the right lung middle lobe was collected and fixed with paraformaldehyde. After dehydration and dealcoholization, the lung tissue was embedded in paraffin. The lung tissue was cut into 4-μm sections and preserved on a slide. Slides were stained with HE. The severity of lung injury was estimated by two independent pathologists who did not participate in this study. The lung histological injury was scored according to the following system: 0, minimum damage; 1, mild damage; 2, moderate damage; 3, severe damage; and 4, maximum damage.

### Apoptosis evaluation

We assessed lung tissue apoptosis by TUNEL staining with commercial kits (Roche Diagnostics GmbH, Science, Mannheim, Germany). The slide with lung tissue section was immersed into proteinase K solution. Then, the slide was rinsed with phosphate-buffered saline (PBS). Afterwards, it was immersed in TUNEL reaction solution. Then, the slide was rinsed three times with PBS and washed with H_2_O_2_ to inhibit the endogenous peroxidase activity. Finally, the slide was immersed in extravidin peroxidase and diaminobenzidine solution. Apoptosis was estimated by an independent pathologist by the brownish staining of the nuclei under a microscope. The apoptosis of endothelium and epithelium was identified and figured out by the independent pathologist to distinguish the apoptosis endothelium and epithelium from inflammatory cells.

### Western blotting

To observe the expression of various proteins in lung tissues, we collected part of the lung tissue and extracted the protein from the lung tissue. The concentrations of proteins were tested with the Bradford assay. We added equivalent protein levels to each the polyacrylamide gel well. After electrophoresis, all the proteins were transferred onto polyvinylidene fluoride membranes (PVDF). The PVDF membrane containing the target protein was cut and blocked with 5% dry milk for 24 h. The PVDF membrane was washed with PBS three times and incubated with endothelin-1, phosphorylated-endothelial nitric oxide synthase (p-eNOS), PKBα, and phosphorylated myosin light chain (p-MLC) (pSer18, Sigma Aldrich). The PVDF membrane was incubated with primary antibodies for 12 h at 4 °C and washed with PBS three times. Then, the membrane was incubated with horseradish peroxidase-linked secondary antibodies (Santa Cruz Biotechnology) for 1 h. Finally, the bands on the PVDF were visualized via enhanced chemiluminescence.

### Cell culture

To investigate the possible mechanism of Ac2-26 on VILI in ARDS, we cultured the human alveolar epithelial cell A549 (American Type Culture Collection, Manassas, CA, United States of America).

To avoid the influence of apoptotic protein extracted from inflammatory cells on the results of Ac2-26 on the epithelium, the human epithelium (A549) was cultured, and received the endotoxin to simulate the ARDS. The A549 cells were obtained from the American Type Culture Collection (ATCC, Manassas, CA, United States of America) and cultured in Dulbecco’s modified Eagles medium (DMEM) combined with Glutamax (Gibco, Grand Island, NY, United States of America), 10% fetal bovine serum (FBS, Gibco), penicillin (100 units/mL) and streptomycin (0.1 mg/mL) (Beyotime, Shanghai, China), under 95% air, 5% CO_2_ atmosphere at 37 °C condition. Then, the cells were washed three time with serum deprived DMEM when the cells were cultured to 80% confluency at a density of 10^4^ cells/cm^2^.

All the cells were divided into four groups: sham group (S), endotoxin group (ARDS), endotoxin/Ac2-26 (ARDS/A) group, and endotoxin/Ac2-26/L-NIO group (ARDS/A/L). The cells in sham did not receive any treatment. The cells in other groups received 15 μg/mL of endotoxin for 4 hours^19^. The cells in ARDS group, ARDS/A group and ARDS/A/L group respectively received the treatment of vehicle, Ac2-26 (0.3 μM), or Ac2-26 (0.3 μM)^10^ and L-NIO 10 μM^20^ in medium.

Four hours after stimulation of endotoxin, the cells were washed and changed the fresh medium. After that, the cell proliferation and viability were detected with the cell counting kit-8 (CCK-8). Briefly, 100 μL 10% CCK-8 solution was added into the 96-well plate for 1 hour. After that, the absorbance of the solution was detected at 450 nm with a microplate reader (Quant Bio Tek Instruments, Winooski, Vermont, United States of America). Moreover, the protein was extracted from epithelium and effect of Ac2-26 on apoptotic regulated protein Bax, Bcl-2 and cleaved caspase-3 were detected and evaluated.

### Apoptosis assay

The Annexin V-FITC/PI apoptosis detection kit (BestBio, Shanghai, China) was purchased from the commercial company and used to investigate the apoptotic cells with flow cytometry (FACScan, Becton Dickinson, United States of America). The epitheliums of four groups were suspended in Annexin-binding buffer and stained with Annexin V-FITC and PI for 30 min in dark room at room temperature. The adherent and floating cells were measured by flow cytometer (Beckman Coulter, United States of America) to distinguish the apoptotic cells (Annexin-V positive and PI-negative) from necrotic cells (Annexin-V and PI-positive).

### Statistical analysis

The primary outcome was the PaO_2_/FiO_2_ ratio after 4 h of ventilation. Our previous study indicated that the PaO_2_/FiO_2_ ratio after 4 h of ventilation was 166 ± 15 mmHg^15^. A power analysis was performed to detect an increase of 50 mmHg in the PaO_2_/FiO_2_ ratio with an alpha error of 0.05 and power of 90%. Based on the power analysis, seven rats were required in each group. To compensate for the potential missed participants, we enrolled eight rats in each group.

The normally distributed data are presented as the mean ± standard deviation (SD), and the skewed data are presented as medians (IQR). The continuous data with normally distributed data were analyzed with two-way repeated analysis of variance (ANOVA). The skewed data were analyzed with a nonparametric Friedman test. When differences were noted, Bonferroni post hoc analysis was performed to identify the source of the difference. All statistical analyses were performed using Statistical Package for the Social Sciences (SPSS) 19.0 for Windows (SPSS, Inc., United States of America). A P value less than 0.05 was considered statistically significant.

## Results

### Ac2-26 improved the alveolo-capillary permeability

**Figure 1:**
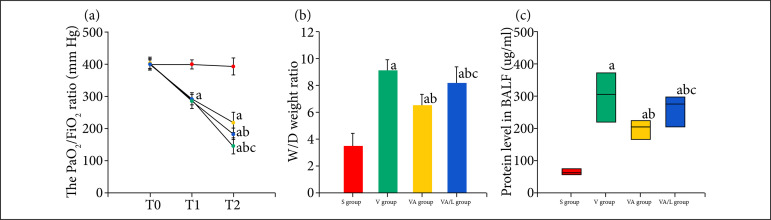
The effect of Ac2-26 on alveolo-capillary permeability. AnexA1 significantly increased the PaO_2_/FiO_2_ ratio and decreased the wet/dry weight ratio and protein concentration in VILI. The Ac2-26-mediated improvements were partly reversed by L-NIO. **(a)** *P* < 0.05 vs. the S group; **(b)** *P* < 0.05 vs. the V group; **(c)** *P* < 0.05 vs. the VA group.

Ac2-26: Annexin A1 peptide; AnexA1: Annexin A1; VILI: ventilator-induced lung injury; L-NIO: N5-(1-iminoethyl)-l-ornithine; S group: sham group; V group: mechanical ventilation group; VA group: mechanical ventilation/Ac2-26 group; VA/L group: mechanical ventilation/Ac2-26/L-NIO group; : S group; : V group; : VA group; : VA/L group).

The PaO_2_/FiO_2_ ratio, total protein concentrations of BALF and lung tissue wet/dry weight ratio were calculated to evaluate the effect of Ac2-26 on alveolo-capillary permeability. We found that the PaO_2_/FiO_2_ ratio significantly decreased after ventilation compared with the one of the S group. The PaO_2_/FiO_2_ ratio was increased, and the wet/dry weight ratio and protein concentrations were decreased by Ac2-26 compared with those of the V group. The Ac2-26 mediated improvement in alveolo-capillary permeability was reversed by the L-NIO treatment in the VAL group compared with the VA group (Fig. 1).

### Ac2-26 reduced endothelial cell injury

**Figure 2:**
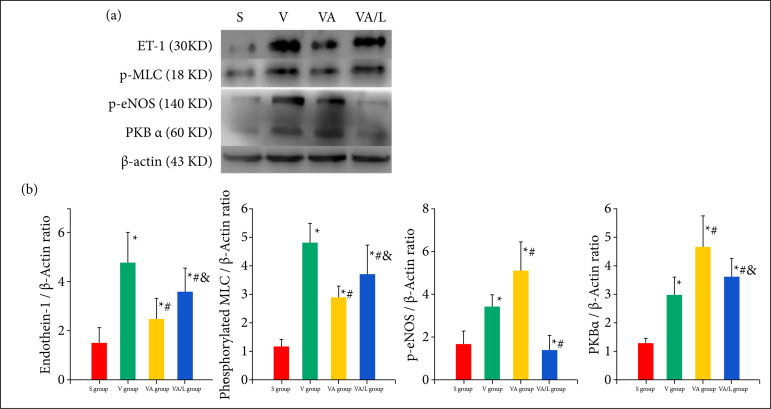
The effect of Ac2-26 on endothelium injury induced by LPS and ventilation. After stimulation of LPS, the ET1, p-MLC, PKBα and p-eNOS were significantly increased. The Ac2-26 reduced the expression of ET-1 and p-MLC, but promoted the expression of PKBα and p-eNOS. **(a)** *P* < 0.05 vs. the S group; **(b)** *P* < 0.05 vs. the L group; **(c)** *P* < 0.05 vs. the LA group.

The expression levels of ET-1 and p-MLC in lung tissues were determined by Western blot analyses. Ac2-26 significantly decreased ET-1 and p-MLC expression in the lung tissue compared with those of the V group. The p-eNOS expression in lung tissue were increased by the Ac2-26, but the promotion of Ac-2-26 on p-eNOS was reduced by the L-NIO. To explore the possible mechanism of Ac2-26 on p-eNOS, we also detected the expression of PKBα in lung tissue. The PKBα was promoted by the Ac2-26 (Fig. 2).

ET-1:endothelin-1; p-MLC: phosphorylated myosin light chain; p-eNOS: phosphorylated-endothelial nitric oxide synthase; PKBα: protein kinase B; LPS: lipopolysaccharide; S group: sham group; V group: mechanical ventilation group; VA group: mechanical ventilation/Ac2-26 group; VA/L group: mechanical ventilation/Ac2-26/L-NIO group; : S group; : V group; : VA group; : VA/L group.

### Ac2-26 inhibited local and systemic inflammation

First, we detected the inflammatory cell counts and cytokine concentrations of BALF to estimate the effect of Ac2-26 on local inflammation.

**Figure 3:**
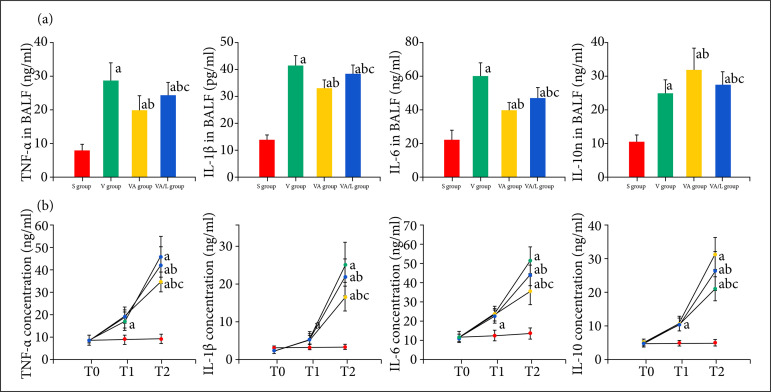
The effect of Ac2-26 on inflammation in VILI. The levels of TNF-α, IL-1β, IL-6 and IL-10 in serum and BALF were determined by ELISAs. The TNF-α, IL-1β, IL-6 and IL-10 in BALF and serum were significantly up-regulated in rats received LPS and ventilation. Compared with V group, the TNF-α, IL-1β, IL-6 were significantly reduced by Ac2-26, but the IL-10 was increased by Ac2-26. The regulation of Ac2-26 was partly reversed by L-NIO. Data are representative images or expressed asthe mean ± standard deviation. **(a)** *P* < 0.05 vs. the S group; **(b)** *P* < 0.05 vs. the V group; **(c)** *P* < 0.05 vs. the VA group.

After 4 h of ventilation, the macrophage and neutrophil counts in BALF were significantly increased in the V and VA groups. The concentrations of elastase and proinflammatory factors, including TNF-α, IL-1β and IL-6, in BALF from the V and VA groups were significantly increased compared with those of the S group. The inflammatory cell count and proinflammatory factors were reduced by Ac2-26 in the VA group compared with the V group. In contrast, the anti-inflammatory IL-10 was increased by Ac2-26. All the changes in these parameters were reversed by the L-NIO compared with those of the VA group (Fig. 3a).

Second, we detected the cytokines in the serum to observe the effect of Ac2-26 on systemic inflammation. Like the results of BALF, the TNF-α, IL-1β and IL-6 levels in serum were significantly upregulated after ventilation, and this upregulation was decreased by Ac2-26. The serum IL-10 was also increased by Ac2-26 compared to that in the V group. The Ac2-26 mediated regulation of serum cytokines was reduced by L-NIO (Fig. 3b).

TNF-α: tumor necrosis factor-α; IL-1β: interleukin-1β; IL-6: interleukin-6; IL-10: interleukin-10; BALF: bronchoalveolar lavage fluid; ELISA: enzyme-linked immunosorbent assay; LPS: lipopolysaccharide; Ac2-26: annexin A1 peptide; L-NIO: N5-(1-iminoethyl)-l-ornithine; S group: sham group; V group: mechanical ventilation group; VA group: mechanical ventilation/Ac2-26 group; VA/L group: mechanical ventilation/Ac2-26/L-NIO group; : S group; : V group; : VA group; : VA/L group.

### Ac2-26 ameliorated oxidative stress in lung tissue

**Figure 4:**
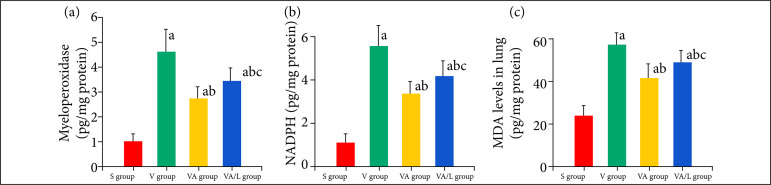
The effect of Ac2-26 on the oxidative stress response in VILI. The MDA levels and MPO and NADPH activities in lung tissues were investigated. Ac2-26 significantly reduced the level of MDA and activity of MPO and NADPH, but L-NIO reversed this effect. **(a)** *P* < 0.05 vs. the S group; **(b)** *P* < 0.05 vs. the V group; **(c)** *P* < 0.05 vs. the VA group.

Ac2-26: annexin A1 peptide; S group: sham group; V group: mechanical ventilation group; VA group: mechanical ventilation/Ac2-26 group; VA/L group: mechanical ventilation/Ac2-26/L-NIO group; : S group; : V group; : VA group; : VA/L group; VILI: ventilator-induced lung injury; MDA: malondialdehyde; MPO: myeloperoxidase; L-NIO: N5-(1-iminoethyl)-l-ornithine.

The levels of MDA, activities of MPO and NADPH were significantly increased in the V and VA groups compared with the S group. The concentrations of MDA, and activities of MPO and NADPH were significantly reduced in the VA group compared with the V group. The inhibition of Ac2-26 on the oxidative stress response was reversed by L-NIO compared with that of the VA group (Fig. 4).

### Effect of Ac2-26 on cell proliferation and viability

**Figure 5:**
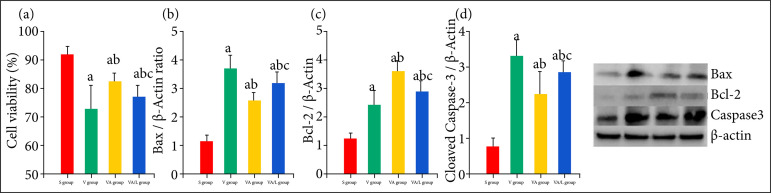
The effect of Ac2-26 on lung histological injury and apoptosis in VILI. **(a)** The lung tissue injury was evaluated with hematoxylin and eosin staining. Ac2-26 significantly reduced lung histological injury, but L-NIO reduced this effect.**(b)** Apoptosis of endothelium and epithelium was estimated by TUNEL staining with independent pathologist. Apoptosis was alleviated by Ac2-26, but this effect was reversed by L-NIO. The cells directed by the arrow were identifiedthe apoptotic cells. **(a)** *P* < 0.05 vs. the S group; **(b)** *P* < 0.05 vs. the V group; **(c)** *P* < 0.05 vs. the VA group.

Ac2-26: annexin A1 peptide; S group: sham group; V group: mechanical ventilation group; VA group: mechanical ventilation/Ac2-26 group; VA/L group: mechanical ventilation/Ac2-26/L-NIO group; : S group; : V group; : VA group; : VA/L group; VILI: ventilator-induced lung injury; L-NIO: N5-(1-iminoethyl)-l-ornithine; VILI: ventilator-induced lung injury.

Compared with S group, the viability of epithelium received the LPS stimulation significantly deteriorated. Compared with L group, the viability of epithelium in LA group was significantly improved, but the protection of Ac2-26 on epithelium was reversed by the L-NIO (Fig. 5a).

### Ac2-26 reduced the histological injury

**Figure 6:**
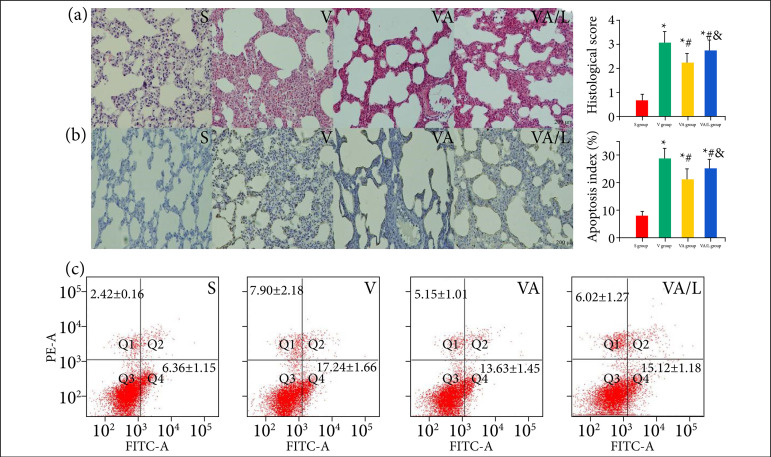
The effect of Ac2-26 on cell proliferation and apoptosis regulated protein in lung tissue and the effect of Ac2-26 on apoptosis of epithelium. **(a)** Compared with S group, the proliferation of epithelium was significantly decreased afterLPS stimulation. Compared with V group, the proliferation of epithelium was improved by the Ac2-26. The protectionof Ac2-26 was partly reduced by the L-NIO. **(b)** The pro-apoptotic protein Bax and cleaved caspase-3 were down-regulated, but the anti-apoptotic protein Bcl-2 was up-regulated by the Ac2-26. The regulation of Ac2-26 was attenuated by the L-NIO. **(a)** *P* < 0.05 vs. the S group; **(b)** *P* < 0.05 vs. the L group; **(c)** *P* < 0.05 vs. the LA group. **(c)** The apoptosis of epithelium wasdetected by the flow cytometer. After stimulation of LPS, the about 18% epithelium was identified apoptosis. Compared with L group, the apoptosis was partly decreased by Ac2-26, but this effect was reversed by the L-NIO (domain ofleft-up is incidence of necrosis, the right-up domain is early apoptosis, and the right-down domain is late apoptosis).

Ac2-26: annexin A1 peptide; S group: sham group; L group: mechanical ventilation group; LA group: mechanical ventilation/Ac2-26 group; VA/L group: mechanical ventilation/Ac2-26/L-NIO group; : S group; : V group; : VA group; : VA/L group; VILI: ventilator-induced lung injury; L-NIO: N5-(1-iminoethyl)-l-ornithine; LPS: lipopolysaccharide.

Compared with the S group, the V and VA groups showed typical lung histological damage, including interstitial edema, alveolar collapse, alveolar breakage, and an increase in the alveolar wall, even hemorrhage, in rats. The lung injury score was significantly reduced by Ac2-26 compared with that of the V group. The effects of Ac2 on lung injury were reduced by L-NIO compared to that of the VA group (Fig. 6a).

### Ac2-26 inhibited the apoptosis

After 4 h of MV, we observed many apoptosis-positive endothelium and epithelium in rats from the V and VA groups compared with those from the S group. Compared with V group, rats in the VA group showed significant reductions in apoptotic cells after treatment with Ac2-26. The effect of Ac2-26 on apoptosis was reversed by the eNOS inhibitor L-NIO (Fig. 6b).

### Effect of Ac2-26 on apoptosis of epithelium

The results of this study indicated that significant apoptosis of epithelium was induced by the endotoxin. The apoptosis of epithelium was inhibited by the Ac2-26 and reversed by L-NIO. The apoptosis and necrosis of epithelium were significantly increased in endotoxin treated group (P<0.05). Compared with ARDS group, the apoptosis and necrosis were decreased by Ac2-26 (P < 0.001), and these effects were partly reduced by the L-NIO–Necrosis: 2.42 ± 0.16 vs. 7.90 ± 2.18 vs. 5.15 ± 1.01 vs. 6.02 ± 1.27; Apoptosis: 6.36 ± 1.15 vs. 17.24 ± 1.66 vs. 13.63 ± 1.54 vs. 15.12 ± 1.18 (Fig. 6c).

We also investigated the effect of Ac2-26 on apoptotic protein in epithelium. The pro-apoptotic protein Bax and apoptotic executive protein cleaved caspase-3 were inhibited, but the anti-apoptotic Bcl-2 was enhanced by the Ac2-26. The effect of Ac2-26 on apoptotic protein was reversed by the L-NIO (Fig. 5b).

## Discussion

The results of this study suggested that Ac2-26 can alleviate VILI in ARDS rats. Ac2-26 significantly improved alveolo-capillary permeability and endothelial function, decreased local and systemic inflammation, reduced the oxidative stress response and inhibited VILI-induced apoptosis in the ARDS rat model. The protection of Ac2-26 on VILI in ARDS was partly inhibited by the L-NIO. Considering the clinical application of Ac2-26 on lung disease, we speculated thatAc2-26 may be another new therapeutic treatment for patients with ARDS that require MV support.

Approximately 5-10% of hospitalized patients were diagnosed with ARDS, and nearly 75% of these patients needed MV support^2^. Despite the application of lung protective strategies, the ARDS-related mortality remained high (27 to 45%)^1^. As an active peptide of AnexA1, Ac2-26 had been indicated to reduce lung injury because of anti-inflammation^9^^,^ 10. Considering the key role of inflammation in VILI and ARDS^1^^,^ 5, we hypothesized that Ac2-26 can reduce the VILI in ARDS. In this study, we injected endotoxin and ventilated the rats to mimic the VILI in ARDS^15^^,^ 16.

After 4 h of ventilation, we found that Ac2-26 ameliorated lung tissue histological injury, reduced the apoptosis, and improved the alveolar-capillary permeability. During the pathology of ARDS and VILI, activation of NF-κB in endothelial and epithelial cells releases chemoattractants and inflammatory factors, including TNF-α, IL-1β, and IL-6, which damage the lung tissue. Under the effect of the chemoattractants, macrophages and neutrophils in peripheral blood are recruited and infiltrate into the injured lung tissue and then release more cytokines into the lung tissue or peripheral blood, further resulting in systemic inflammatory response syndrome^6^^,^ 8.

In this study, Ac2-26 significantly reduced the pro-inflammatory factors and chemoattractants. Moreover, Ac2-26 also reduced the infiltration of macrophages and neutrophils and decreased the levels of elastase, TNF-α, IL-1β, and IL-6 but increased the anti-inflammatory cytokine IL-10 in BALF. TNF-α and IL-1β play pivotal roles in VILI and ARDS^21^. During ARDS and VILI, the cytokines TNF-α, IL-1β and IL-6 directly injure the lung endothelial and epithelial cells and aggravate lung inflammation. The effect of Ac2-26 on local inflammation may be due to the inhibition of NF-κB^22^ and the decreased secretion of ICAM-1 and IL-8, which can promote the migration of macrophages and neutrophils of the peripheral blood into the lung tissue^23^^,^ 24. Furthermore, Ac2-26 reduced inflammation via promotion of IL-10, which can inhibit aggravation of TNF-α, IL-1β, and IL-6 in lung injury^25^.

Inflammation induced by both LPS and ventilation can damage endothelium and express high levels of ET-1^26^, and the ET-1 can enhance the iNOS expression in endothelium^27^. The iNOS not only produced the reactive oxidative species (ROS), but also promoted the production of inflammatory cytokines. Contrast to iNOS, the eNOS had been indicated to protect the endothelium injury and reduce the lung injury^11^. Moreover, injured endothelium would result in the myosin light chain (MLC) phosphorylation, which disrupt the endothelial barrier and enhance the lung edema during the process of acute lung injury^28^ and further contributing to the lung edema^29^. In this study, the Ac2-26 treatment significantly decreased the levels of ET-1 and phosphorylated MLC in the lung tissue, but reinforced the expression of p-eNOS. These results indicated that Ac2-26 significantly ameliorated the endothelium injury in VILI.

In addition to inflammation, the oxidative stress response also plays an important role^30^^,^ 31 in VILI. Activated neutrophils convert oxygen into hydrogen peroxide and superoxide anions through NADPH oxidase^32^^,^ 33. MDA, which is the final product of this response, directly indicates the severity of the oxidative stress response^34^. In this study, we found that Ac2-26 significantly reduced the MDA level, and this result suggested that Ac2-26 inhibited the oxidative stress response. Also, we found Ac2-26 decreased the activity of NADPH and MPO, which is a marker of neutrophils, and the severity of the oxidative stress response^35^. Therefore, we speculated that anti-oxidative effect of Ac2-26 mainly due to the MPO and NADPH pathway^36^.

During VILI in ARDS, proinflammatory factors, such as TNF-α and ROS release from the injured endothelium and epithelium both resulted to apoptosis by activating the intrinsic and extrinsic apoptosis pathways. In this study, we found that Ac2-26 significantly inhibited apoptosis of endothelium and epithelium, and the anti-apoptotic effect of Ac2-26 maybe with the inhibition of Ac2-26 on TNF-α and oxidative stress.

In vivo part of this study, we did not detect the apoptotic regulated protein, because the protein sample was mixed from lung constituent tissue and inflammatory cells, as we known that the Ac2-26 promoted the apoptosis on inflammatory cells. Therefore, we cultured and activated the epithelium with LPS, and we found that Ac2-26 significantly improved the viability and decreased the apoptosis of epithelium.

This study indicated that the protection of Ac2-26 on VILI not only depended on inhibition on inflammatory cells, but also attributed to directly protection on the epithelium. We also found that Ac2-26 inhibited the pro-apoptotic protein Bax and apoptosis executor cleaved-caspase 3, but enhanced the anti-apoptotic protein Bcl-2 in epithelium. Bax is an important pro-apoptotic protein, whereas Bcl-2 is an anti-apoptotic protein that can prevent Bax activation. An increase or decrease in apoptosis has been shown to primarily depend on the ratio of Bax to Bcl-2^37^. Under pro-apoptotic signaling, caspase-3 is activated (*i.e.*, cleaved) and cuts DNA to mediate cellular apoptosis. The results showed that Ac2-26 significantly decreased the pro-apoptotic protein Bax and the apoptotic-executor cleaved caspase-3, but increased the expression of the anti-apoptotic protein Bcl-2.

There were investigations applied the special device such as Flexcell Strain Unit to simulate the lung injury induced by ventilator using epithelium. However, we cannot simulate this epithelial injury in vitro part because shortage of this equipment. In our future study, we will perform the cell model to investigate the mechanism of Ac2-26 on VILI using similar equipment. Moreover, in this study, we did not observe the effect of Ac2-26 on endothelial cells, because of shortage of funding.

## Conclusion

Ac2-26 mitigates VILI in ARDS rats and partly depended on the endothelial nitric oxide synthase pathway.

## References

[1] Force ADT, Ranieri VM, Rubenfeld GD, Thompson BT, Ferguson ND, Caldwell E, Fan E, Camporota L, Slutsky AS. (2012). Acute respiratory distress syndrome: the Berlin Definition. JAMA.

[2] Bellani G, Laffey JG, Pham T, Fan E, Brochard L, Esteban A, Gattinoni L, van Haren, Larsson A, McAuley DF, Ranieri M, Rubenfeld G, Thompson BT, Wrigge H, Slutsky AS, Pesenti A, Investigators LS, Group ET. (2016). Epidemiology, patterns of care, and mortality for patients with acute respiratory distress syndrome in intensive care units in 50 countries. JAMA.

[3] Esteban A, Anzueto A, Alia I, Gordo F, Apezteguia C, Palizas F, Cide D, Goldwaser R, Soto L, Bugedo G, Rodrigo C, Pimentel J, Raimondi G, Tobin MJ (2000). How is mechanical ventilation employed in the intensive care unit? An international utilization review. Am J Resp Crit Care Med.

[4] Slutsky AS, Ranieri VM. (2013). Ventilator-induced lung injury. N Engl J Med.

[5] Nakamura T, Malloy J, McCaig L, Yao LJ, Joseph M, Lewis J, Veldhuizen R. (2001). Mechanical ventilation of isolated septic rat lungs: effects on surfactant and inflammatory cytokines. J Appl Physiol..

[6] Halbertsma FJ, Vaneker M, Scheffer GJ, van der (2005). Cytokines and biotrauma in ventilator-induced lung injury: a critical review of the literature. Neth J Med..

[7] Kneyber MC, Zhang H, Slutsky AS. (2014). Ventilator-induced lung injury. Similarity and differences between children and adults. Am J Resp Crit Care Med.

[8] Held HD, Boettcher S, Hamann L, Uhlig S. (2001). Ventilation-induced chemokine and cytokine release is associated with activation of nuclear factor-kappaB and is blocked by steroids. Am J Resp Crit Care Med..

[9] Guido BC, Zanatelli M, Tavares-de-Lima W, Oliani SM, Damazo AS. (2013). Annexin-A1 peptide down-regulates the leukocyte recruitment and up-regulates interleukin-10 release into lung after intestinal ischemia-reperfusion in mice. J Inflamm (Lond).

[10] Liao WI, Wu SY, Wu GC, Pao HP, Tang SE, Huang KL, Chu SJ. (2017). Ac2-26, an annexin A1 peptide, attenuates ischemia-reperfusion-induced acute lung injury. Int J Mol Sci..

[11] Takenaka K, Nishimura Y, Nishiuma T, Sakashita A, Yamashita T, Kobayashi K, Satouchi M, Ishida T, Kawashima S, Yokoyama M. (2006). Ventilator-induced lung injury is reduced in transgenic mice that overexpress endothelial nitric oxide synthase. Am J Physiol Lung Cell Mol Physiol.

[12] Yamashita T, Kawashima S, Ohashi Y, Ozaki M, Ueyama T, Ishida T, Inoue N, Hirata K, Akita H, Yokoyama M. (2000). Resistance to endotoxin shock in transgenic mice overexpressing endothelial nitric oxide synthase. Circulation.

[13] Cui Y, Yang S. (2018). Overexpression of Annexin A1 protects against benzo[a]pyreneinduced bronchial epithelium injury. Mol Med Rep..

[14] Purvis GSD, Chiazza F, Chen J, Azevedo-Loiola R, Martin L, Kusters DHM, Reutelingsperger C, Fountoulakis N, Gnudi L, Yaqoob MM, Collino M, Thiemermann C, Solito E. (2018). Annexin A1 attenuates microvascular complications through restoration of Akt signalling in a murine model of type 1 diabetes. Diabetologia.

[15] Gao W, Ju YN. (2016). Budesonide attenuates ventilator-induced lung injury in a rat model of inflammatory acute respiratory distress syndrome. Arch Med Res.

[16] Ju YN, Yu KJ, Wang GN. (2016). Budesonide ameliorates lung injury induced by large volume ventilation. BMC Pulm Med.

[17] Cunha EE, Oliani SM, Damazo AS. (2012). Effect of annexin-A1 peptide treatment during lung inflammation induced by lipopolysaccharide. Pulm Pharmacol Ther.

[18] Greco R, Demartini C, Zanaboni AM, Blandini F, Amantea D, Tassorelli C. (2018). Endothelial nitric oxide synthase inhibition triggers inflammatory responses in the brain of male rats exposed to ischemia-reperfusion injury. J Neurosci Res..

[19] Liu Q, Yang H, Xu S, Sun X (2018). Downregulation of p300 alleviates LPS-induced inflammatory injuries through regulation of RhoA/ROCK/NF-kappaB pathways in A549 cells. Biomed Pharmacother.

[20] Tsai KL, Huang YH, Kao CL, Yang DM, Lee HC, Chou HY, Chen YC, Chiou GY, Chen LH, Yang YP, Chiu TH, Tsai CS, Ou HC, Chiou SH. (2012). A novel mechanism of coenzyme Q10 protects against human endothelial cells from oxidative stress-induced injury by modulating NO-related pathways. J Nutr Biochem..

[21] Wilson MR, Choudhury S, Takata M. (2005). Pulmonary inflammation induced by high-stretch ventilation is mediated by tumor necrosis factor signaling in mice. Am J Physiol Lung Cell Mol Physiol.

[22] Yang YH, Morand E, Leech M. (2013). Annexin A1: potential for glucocorticoid sparing in RA. Nat Rev Rheumatol.

[23] Miyao N, Suzuki Y, Takeshita K, Kudo H, Ishii M, Hiraoka R, Nishio K, Tamatani T, Sakamoto S, Suematsu M, Tsumura H, Ishizaka A, Yamaguchi K. (2006). Various adhesion molecules impair microvascular leukocyte kinetics in ventilator-induced lung injury. A Am J Physiol Lung Cell Mol Physiol..

[24] Li LF, Liao SK, Lee CH, Huang CC, Quinn DA. (2007). Involvement of Akt and endothelial nitric oxide synthase in ventilation-induced neutrophil infiltration: a prospective, controlled animal experiment. Crit Care..

[25] Opal SM, DePalo VA. (2000). Anti-inflammatory cytokines. Chest.

[26] Ju YN, Gong J, Wang XT, Zhu JL, Gao W. (2018). Endothelial colony-forming cells attenuate ventilator-induced lung injury in rats with acute respiratory distress syndrome. Arch Med Res..

[27] Shaw MJ, Shennib H, Bousette N, Ohlstein EH, Giaid A. (2001). Effect of endothelin receptor antagonist on lung allograft apoptosis and NOSII expression. Ann Thorac Surg.

[28] Dudek SM, Garcia JG. (2001). Cytoskeletal regulation of pulmonary vascular permeability. J Appl Physiol (1985).

[29] Rossi JL, Velentza AV, Steinhorn DM, Watterson DM, Wainwright MS (2007). MLCK210 gene knockout or kinase inhibition preserves lung function following endotoxin-induced lung injury in mice. Am J Physiol Lung Cell Mol Physiol.

[30] Mahmoodpoor A, Hamishehkar H, Shadvar K, Ostadi Z, Sanaie S, Saghaleini SH, Nader ND. (2019). The effect of intravenous selenium on oxidative stress in critically Ill patients with acute respiratory distress syndrome. Immunol Invest.

[31] Huang CS, Kawamura T, Lee S, Tochigi N, Shigemura N, Buchholz BM, Kloke JD, Billiar TR, Toyoda Y, Nakao A. (2010). Hydrogen inhalation ameliorates ventilator-induced lung injury. Crit Care.

[32] Sato K, Kadiiska MB, Ghio AJ, Corbett J, Fann YC, Holland SM, Thurman RG, Mason RP. (2002). In vivo lipid-derived free radical formation by NADPH oxidase in acute lung injury induced by lipopolysaccharide: a model for ARDS. FASEB J..

[33] Liu YY, Li LF, Fu JY, Kao KC, Huang CC, Chien Y, Liao YW, Chiou SH, Chang YL. (2014). Induced pluripotent stem cell therapy ameliorates hyperoxia-augmented ventilator-induced lung injury through suppressing the Src pathway. PloS One.

[34] Liu KX, Wu WK, He W, Liu CL. (2007). Ginkgo biloba extract (EGb 761) attenuates lung injury induced by intestinal ischemia/reperfusion in rats: roles of oxidative stress and nitric oxide. World J Gastroenterol.

[35] Bradley PP, Priebat DA, Christensen RD, Rothstein G. (1982). Measurement of cutaneous inflammation: estimation of neutrophil content with an enzyme marker. J Invest Dermatol.

[36] Leoni G, Alam A, Neumann PA, Lambeth JD, Cheng G, McCoy J, Hilgarth RS, Kundu K, Murthy N, Kusters D, Reutelingsperger C, Perretti M, Parkos CA, Neish AS, Nusrat A. (2013). Annexin A1, formyl peptide receptor, and NOX1 orchestrate epithelial repair. J Clin Invest.

[37] Li L, Wu W, Huang W, Hu G, Yuan W, Li W. (2013). NF-kappaB RNAi decreases the Bax/Bcl-2 ratio and inhibits TNF-alpha-induced apoptosis in human alveolar epithelial cells. Inflamm Res..

